# Angiotensins and Huntington’s Disease: A Study on Immortalized Progenitor Striatal Cell Lines

**DOI:** 10.3389/fendo.2017.00108

**Published:** 2017-05-24

**Authors:** Walmor C. De Mello, Yamil Gerena, Sylvette Ayala-Peña

**Affiliations:** ^1^Department of Pharmacology and Toxicology, School of Medicine, Medical Sciences Campus, University of Puerto Rico, San Juan, PR, USA

**Keywords:** angiotensin II, angiotensin (1-7), Huntington’s disease, AT1 receptor, potassium currents, mouse striatal cell lines

## Abstract

Neurons from mouse models of Huntington’s disease (HD) exhibit altered electrophysiological properties, potentially contributing to neuronal dysfunction and neurodegeneration. The renin–angiotensin system (RAS) is a potential contributor to the pathophysiology of neurodegenerative diseases. However, the role of angiotensin II (Ang II) and angiotensin (1-7) has not been characterized in HD. We investigated the influence of Ang II and angiotensin (1-7) on total potassium current using immortalized progenitor mutant huntingtin-expressing (Q111) and wild-type (Q7) cell lines. Measurements of potassium current were performed using the whole cell configuration of pCLAMP. The results showed that (1) the effect of Ang II administered to the bath caused a negligible effect on potassium current in mutant Q111 cells compared with wild-type Q7 cells and that intracellular administration of Ang II reduced the potassium current in wild type but not in mutant cells; (2) the small effect of Ang II was abolished by losartan; (3) intracellular administration of Ang II performed in mutant huntingtin-expressing Q111 cells revealed a negligible effect of the peptide on potassium current; (4) flow cytometer analysis indicated a low expression of Ang II AT1 receptors in mutant Q111 cells; (5) mutant huntingtin-expressing striatal cells are highly sensitive to Ang (1-7) and that the effect of Ang (1-7) is related to the activation of Mas receptors. In conclusion, mutant huntingtin-expressing cells showed a negligible effect of Ang II on potassium current, a result probably due to the reduced expression of AT1 receptors at the surface cell membrane. In contrast, administration of Ang (1-7) to the bath showed a significant decline of the potassium current in mutant cells, an effect dependent on the activation of Mas receptors. Ang II had an intracrine effect in wild-type cells and Ang (1-7) exerted a significant effect in mutant huntingtin-expressing striatal cells.

## Introduction

Huntington’s disease (HD) is a devastating, progressive neurodegenerative disease with autosomal dominant inheritance, characterized by involuntary movements, motor dysfunction, cognitive decline, and behavioral irregularities (1). HD is caused by an expanded CAG repeat (≥39) in the gene encoding the protein huntingtin. The expanded allele expresses a mutant form of huntingtin with acquired toxic properties (2). Expression of mutant huntingtin in the brain causes neuronal loss mainly in the striatum and cerebral cortex (3, 4). The mechanisms by which mutant huntingtin leads to pathology remain unclear. In neurons, potassium currents affect many functions including action potential frequency and neurotransmitter release, cell proliferation, and apoptosis (5). Interestingly, the electrophysiological properties of neurons are altered in HD mouse models, potentially contributing to neuronal dysfunction and neurodegeneration (6–8).

Evidence from studies in HD animal models links the renin–angiotensin system (RAS) to the pathophysiology of neurodegenerative diseases (9, 10). The brain RAS is a potential contributor to neurodegeneration and has been suggested to play a role in the etiology and progression of Alzheimer’s disease (AD) (11) and Parkinson’s disease (PD) (12). Decreased angiotensin II (Ang II) receptor binding in the substantia nigra and striatum in the brain of patients with PD has been described, and evidence is available that neurons are able to synthesize angiotensinogen in ventrolateral medulla (13), thalamus, hypothalamus, and brain stem (13–15) and that Ang II is found in supraoptic nuclei and hypothalamus (16). Of particular interest is the observation that Ang II has been localized in nerve terminals (17) raising the possibility that the peptide is a neurotransmitter. Other studies revealed the presence of renin protein and mRNA in astrocytes (18), but the precise meaning of these findings is not known. Moreover, angiotensin converting enzyme (ACE) inhibitors and AngII antagonists prevent cognitive decline in a chemically induced mouse model of HD (19) and in models of AD (20).

Over the last two decades, knowledge on the pathophysiology and molecular biology of HD has significantly extended, and the contribution of non-CNS tissues to the pathogenesis and clinical symptomatology is increasingly recognized. Interestingly, an important role of the immune system has been found in HD. High titers of T cell activating autoantibodies against Ang II type 1 receptors (AT1R) are present in HD patients as compared to healthy controls (21). Higher anti-AT1R antibody titers associated with early onset of disease and significantly correlated with disease duration and disease burden score (21). Moreover, activation of the peripheral immune system and in particular an upregulation of innate immune responses including microglia activation has been repeatedly reported in HD patients (22–24). Interestingly, the activity of ACE is significantly reduced in the caudate putamen of HD patients (25). However, no information is available showing the effects of angiotensins in HD. In this study, we investigated the effects of Ang II and Ang (1-7) in HD using immortalized progenitor mutant huntingtin-expressing Q111 and wild-type Q7 mouse striatal cell lines.

## Materials and Methods

### Cell Culture

Immortalized progenitor cell lines STHdhQ111 mutant (Q111) and STHdhQ7 wild type (WT; Q7) were derived from striatal neurons from HdhQ111/Q111 and HdhQ7/Q7 mice (expressing 111 and 7 glutamine repeats, respectively) and were kindly provided by Dr. Marcy McDonald, Massachusetts General Hospital. Cells were cultured in Dulbecco’s modified Eagle medium supplemented with10% FBS, 100 U/ml penicillin, 100 mg/ml streptomycin, 2mM l-glutamine, and 400 mg/ml G418. Cells were grown at 33°C in a 5% CO_2_ incubator. Cells with passages lower than 14 were used in all experiments.

### Electrophysiology

Electrophysiological recordings of total potassium current were made using whole cell patch-clamp procedures in the voltage-clamp mode. Experiments were performed at room temperature with an Axopatch 200B amplifier and a Digidata 1400B interface (Molecular Devices, CA). Data acquisition and analyses were performed using pClamp 10. Cells were bathed in modified Tyrode’s solution containing (in millimolars) 137 NaCl, 5.4 KCl, 1.35 CaCl_2_.MgSO_4_.7 H_2_O, 0.3 NaH_2_PO_4_, 10 HEPES, 5 dextrose, pH adjusted to 7.4. Neurons in the culture dish (volume 2 ml) were superfused at a rate of 1–2 ml/min. The patch electrodes had resistances of 2–4 MΩ when filled with an internal pipette solution containing (mM): 130 KCl, 2 MgCl_2_, 0.25 CaCl_2_, 3 ATP, 5 dextrose, 5 EGTA, and 10 HEPES, pH adjusted to 7.2 with KOH. Series resistance was compensated (50%) with Axopatch 200B compensation circuitry. Standard recording conditions for total outward K current consisted in holding the membrane potential to −80 mV and applying depolarizing steps of 10 mV for 300 ms.

### Membrane Levels of AT1 Receptor

Q7 (WT) and Q111 (mutant huntingtin-expressing) cells (1 × 10^6^ cells) were incubated with an anti-AT1 receptor primary antibody (5 μg; EMD Millipore, MA, USA) for 1 h at 4°C. The cells were then washed two times with 1× PBS and then incubated with an FITC-secondary antibody (1:500) for an additional hour at 4°C. Samples were analyzed by flow cytometry. Unlabeled cells were used to determine the autofluorescence level.

### Flow Cytometry

All flow cytometric analyses were carried out using a four-color flow cytometer (FACSCalibur, BD Biosciences, San Jose, CA, USA) equipped with a 488 nm argon-ion laser and a 635 nm red-diode laser. Cell size and granularity were determined on FSC versus SSC dot plots. Emission for FITC fluorescence from AT1 receptor was measured in the FL1 photomultiplier through a 530/30 nm bandpass filter. A total of 20,000 events were analyzed for each sample, and list-mode files were collected using Cell Quest software 3.3 (BD Biosciences, San Jose, CA, USA) and analyzed using the FlowJo software vX.0.7 (BD Biosciences, San Jose, CA, USA). The mean fluorescence intensities of A7 cells and Q111 mutants were obtained from the flow cytometric histogram plots.

### Drugs

Angiotensin II, angiotensin (1-7), A779, and Bis-1 were form Sigma Chemical Co., St. Louis, MO, USA.

### Statistical Analysis

Data are expressed as mean ± SEM. Student’s *t*-test was used. Differences were considered significant when *p* < 0.05.

## Results

### Poor Sensitivity of Mutant Q111 Huntingtin-Expressing Striatal Cells to Extracellular Ang II

**Figure 1:**
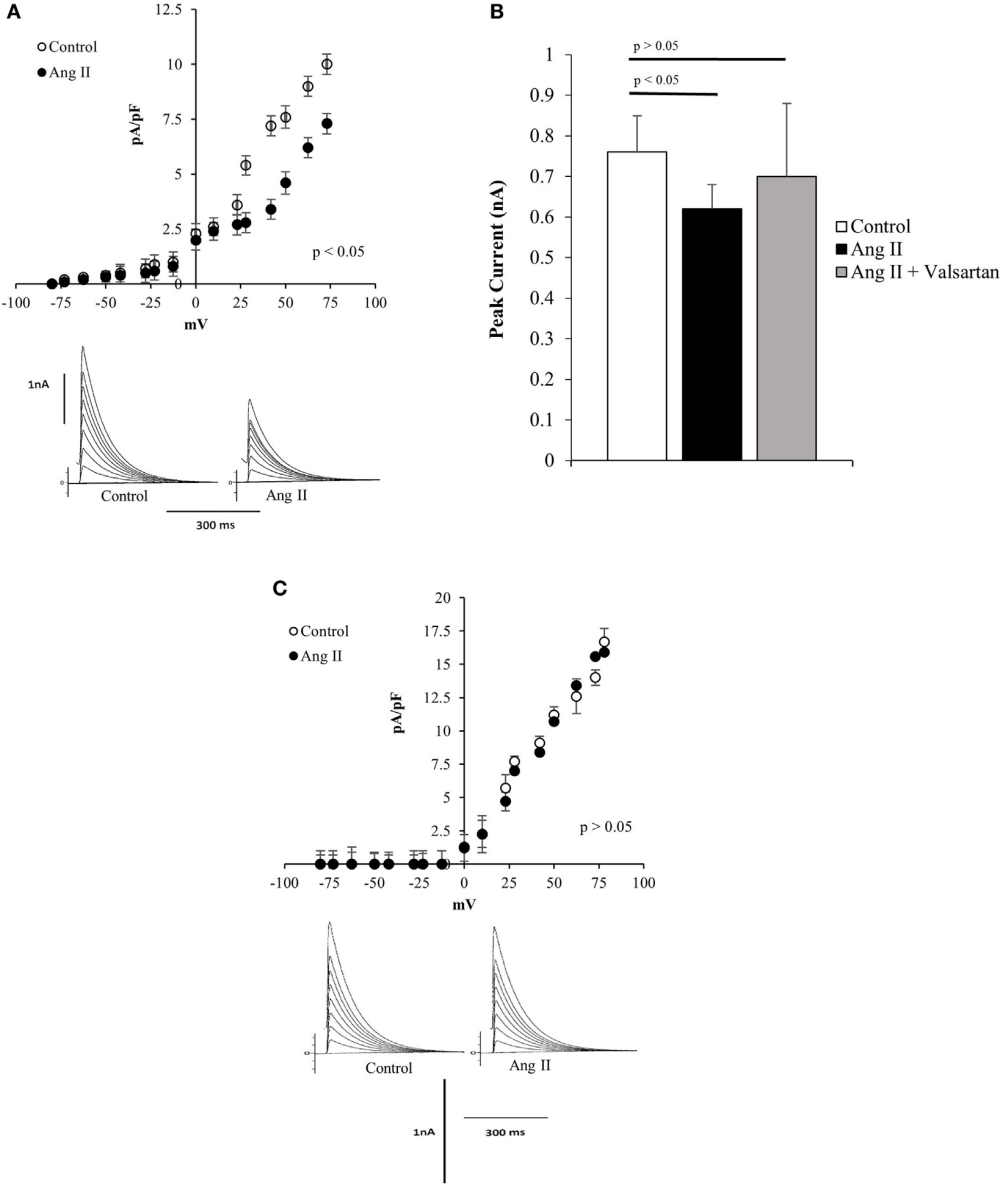
**(A)** Top—Current/voltage curves showing the decline of total potassium current in normal Q7 cells elicited by extracellular administration of angiotensin II (Ang II) (100 nM) to the bath. Each point is the average of 35 cells. Vertical line at each point represents the SEM (*p* < 0.05). Bottom—Potassium currents recoded from single Q7 cells before (control) and after the administration of Ang II (100 nM) to the bath. Vertical calibration represents 1 nA; horizontal calibration represents 300 ms. Holding potential −80 mV. **(B)** Influence of valsartan (10^−9^M) on the effect of Ang II (100 nM) on peak potassium current elicited by a depolarizing pulse from the holding potential −80 to 0 mV. Each bar is the average from 25 Q7 cells. Vertical line at each bar represents the SEM. **(C)** Top—Current/voltage curve showing the lack of action of Ang II (100 nM) on several Q111 mutant cells. Each point is the average from 36 cells. Vertical line at each bar represents SEM (*p* > 0.05). Bottom—Lack of action of Ang II (100 nM) administered to the bath on total potassium current recorded from Q111 single cell before (control) and after the administration of Ang II to the bath. Vertical calibration represents 1 nA; horizontal calibration represents 300 ms.

To investigate the effect of Ang II on potassium current, the peptide was administered to the bath, and measurements of potassium current were performed before and after the administration of the peptide. As shown in Figure 1A, Ang II (100 nM) decreases the potassium current in normal Q7 striatal cells, and concurrently with the change of potassium current, the resting potential was found to be increased by about 8 ± 2.4 mV (*n* = 22) (*p* < 0.05). The effect of the peptide on potassium current was inhibited by valsartan (10^−9^M) as shown in Figure 1B. We found important to investigate if the mutant Q111 cells are also less sensitive to Ang II. Experiments performed on these cells showed that Ang II (100 nM) added to the bath had a negligible effect on potassium current as illustrated in Figure 1C.

### Reduced Expression of AT1 Receptors in Mutant Q111 Cells

**Figure 2:**
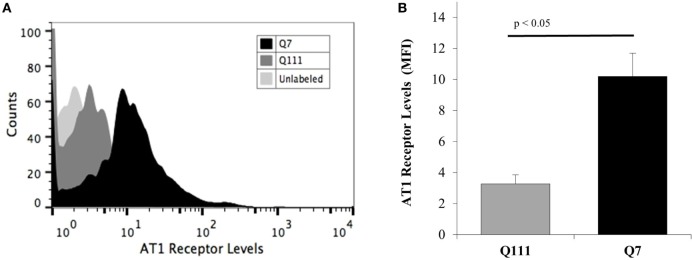
**Membrane levels of AT1 receptor in A7 cells and Q111 mutant cells**. Cells were stained for membrane AT1 receptor using an anti-AT1 receptor primary antibody, and their fluorescence intensity was analyzed by flow cytometry using an FITC-secondary antibody. **(A)** Representative flow cytometric histograms show the differences in the fluorescence intensities of membrane AT1 receptor levels from Q7 (dark histogram) and Q111 mutant cells (dark gray histogram). The light gray histogram represents the unlabeled cells. **(B)** Graph shows the significant differences (*p* < 0.05) between the AT1 receptor levels of Q7 (dark bar) and Q111 mutant cells (gray bar) obtained from the mean fluorescence intensities (MFI) of the histograms. Error bars represent the SD.

To investigate if the reduced effect of Ang II on potassium current in Q111 cells was related to a decreased expression of AT1R, flow cytometry analysis was performed in Q7 and Q111 cells. The results revealed a significant 67% reduction of AT1R levels in Q111 mutant huntingtin-expressing cells when compared with normal Q7 controls (see Figure 2).

### Intracellular Ang II and Potassium Current

**Figure 3:**
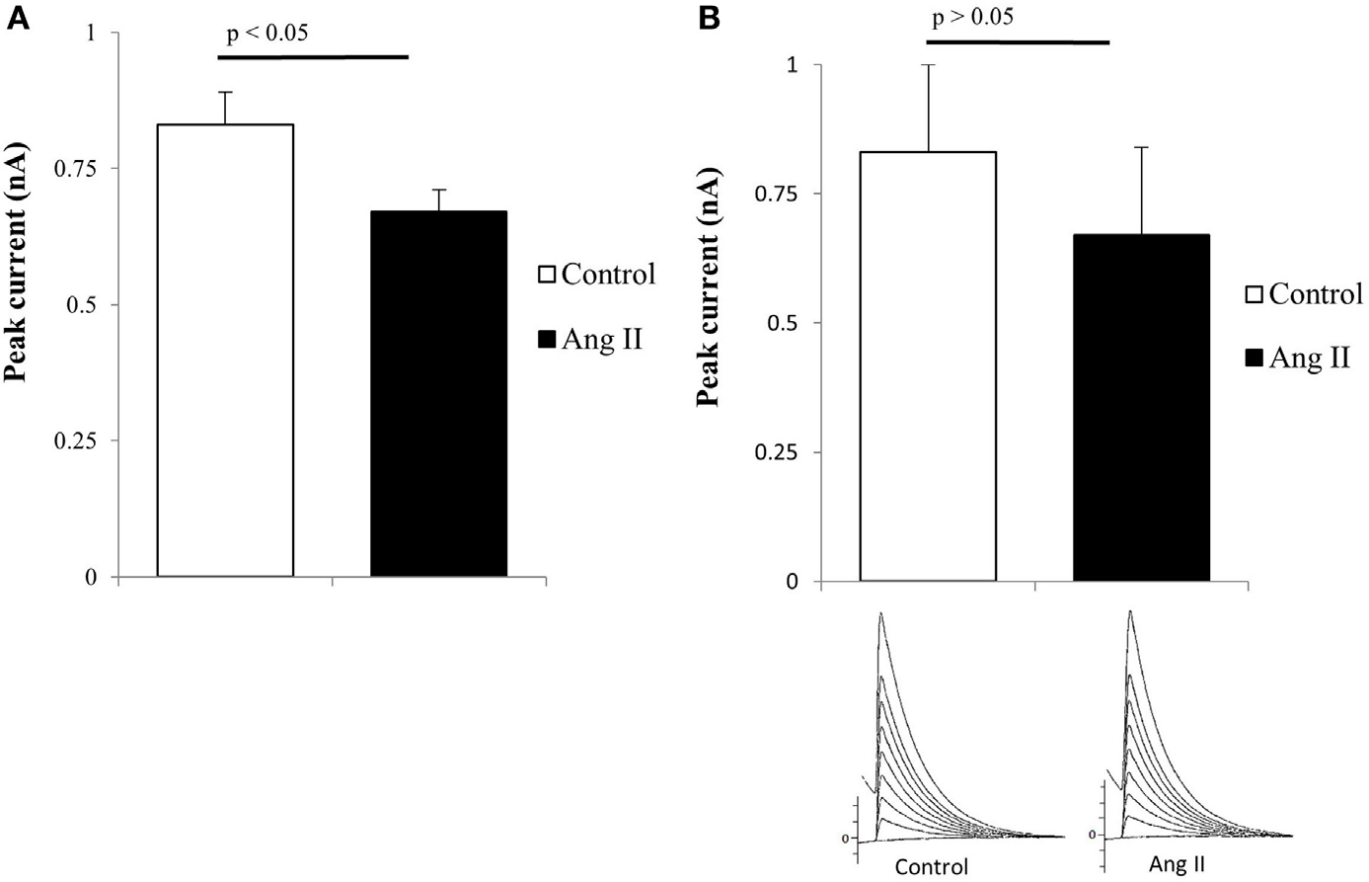
**(A)** Decrease of total potassium current caused by intracellular administration of angiotensin II (Ang II) (100 nM) in normal Q7 cells. Each bar is the average from 32 cells. Vertical line at each bar represents the SEM (*p* < 0.05). **(B)** Negligible effect of intracellular administration of Ang II (100 nM) in Q111 mutant cells. Each bar is the average from 36 cells. The vertical line at each bar represents the SEM (*p* > 0.05).

Previous findings indicated that intracellular Ang II changes the inward calcium current and cell communication in the heart and other tissues (26–34) and that AT1 receptors were found intracellularly near mitochondria (35). These observations indicate that endogenous or internalized Ang II can alter mitochondrial function including calcium release or enhancement of oxidative stress (35–38). It is then important to investigate if intracellular Ang II has an effect on potassium current in mutant huntingtin-expressing Q111 cells. To investigate this possibility, the peptide was added to the pipette solution and dialyzed into the cells using the patch-clamp technique. Figure 3A shows that in wild-type Q7 cells, the intracellular administration of the peptide reduces the potassium current while in mutant Q111 cells the same concentration of Ang II caused a negligible effect on the potassium current (see Figure 3B).

### Q111 Mutant Cells Are Highly Sensitive to Ang (1-7)

**Figure 4:**
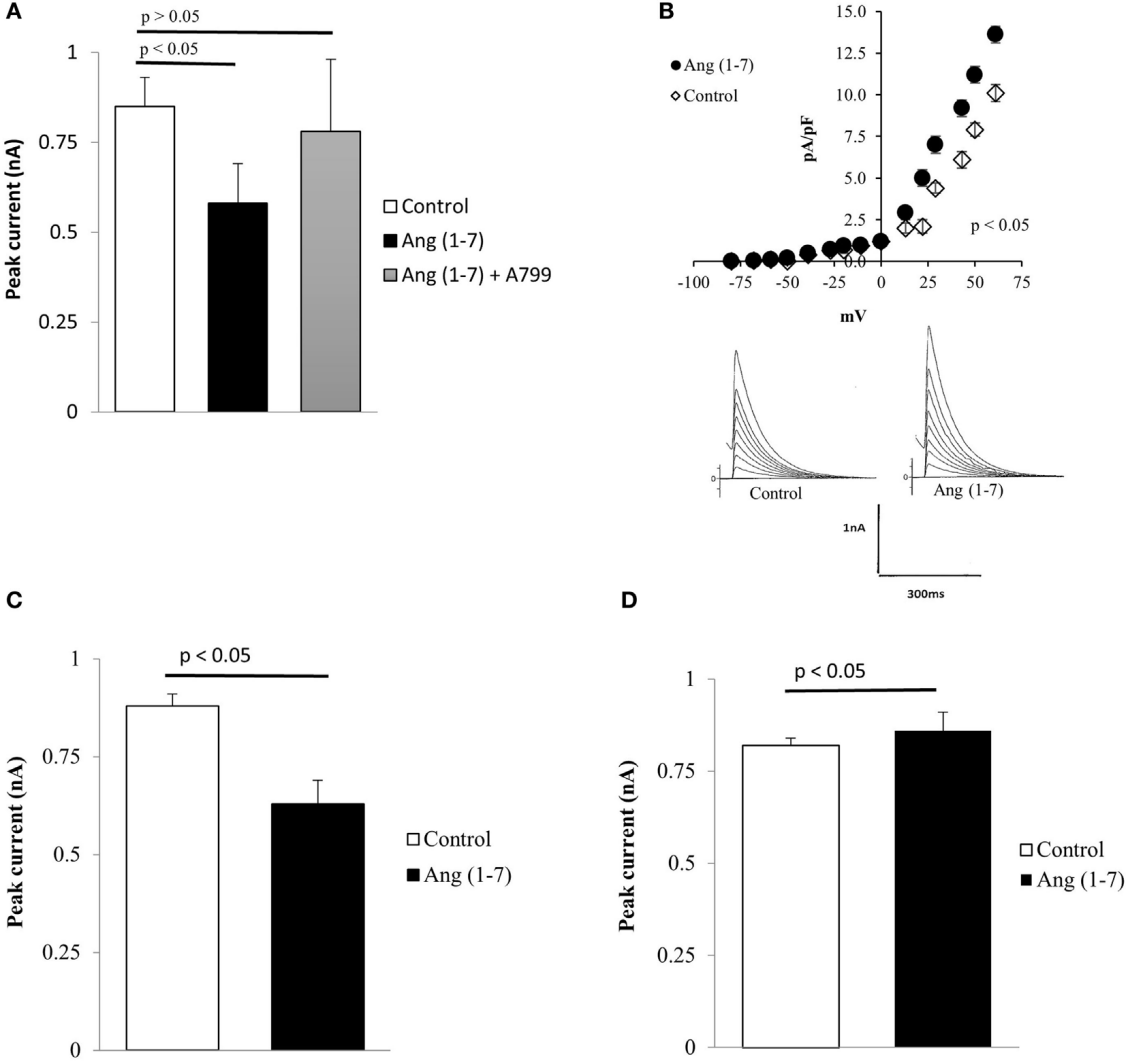
**(A)** Inhibition of the effect of Ang (1-7) elicited by A-799 (10^−8^M) administered to the bath. Each bar is the average from 24 cells. Vertical line at each point represents the SEM (*p* < 0.05). **(B)** Increase of total potassium current caused by extracellular administration of Ang (1-7) (100 nM) in Q7 cells. Top—current–voltage curves showing the effect of the heptapeptide in several Q7 cells. Each point is the average from 33 cells. Vertical calibration at each point represents the SEM (*p* < 0.05). Bottom—effect of the heptapeptide in single cells before (control) and after the administration of Ang (1-7) to the bath. Vertical calibration represents 1 nA; horizontal calibration represents 300 ms. **(C)** Decrease of total potassium current caused by intracellular administration of Ang (1-7) (100 nM) in mutant Q111 cells. Each bar is the average from 28 cells. Vertical line at each bar represents the SEM (*p* < 0.05). **(D)** Small increase of total potassium current recorded from normal Q7 cells seen after the intracellular administration of Ang (1-7) (100 nM). Each bar—average from 29 cells. Vertical line at each bar represents the SEM (*p* < 0.05).

Evidence has been provided that the activation of the ACE2/Ang (1-7)/Mas axis counteracts many effects of Ang II in the cardiovascular and central nervous systems (27). To investigate the influence of Ang (1-7) in HD, the heptapeptide was administered to the extracellular fluid, and its effect on potassium current was investigated in immortalized Q111 mutant huntingtin-expressing and wild-type Q7 mouse striatal cells. As shown in Figure 4A, the administration of Ang (1-7) (10^−9^M) to the bath elicited a decrease in the total potassium current in Q111 mutant cells. Interestingly, this effect is related to the activation of Mas receptors because A-799, which is a Mas receptor antagonist, added to the bath, abolished the effect of the heptapeptide (Figure 4A). In wild-type Q7 cells, the same dose of the heptapeptide elicited a small but statistically significant increase of potassium current as shown in Figure 4B.

### Intracrine Effect of Ang (1-7) on Q111 and Q7 Cells

The presence of Mas receptors and Ang (1-7) in the cell nucleus (35, 39) raises the possibility that the heptapeptide plays an important role on cellular functions. We investigated the possible intracrine effect of Ang (1-7) on potassium current in both Q111 and Q7 cells. As it can be seen in Figure 4C, Ang (1-7) significantly reduced the potassium current in Q111 mutant cells. Comparative experiments performed on wild-type Q7 cells indicated a small but statistically significant increment of potassium current (Figure 4D).

## Discussion

The present results demonstrate for the first time that mutant huntingtin-expressing striatal cells are highly sensitive to Ang (1-7), a conclusion supported by the finding that the decrease of total potassium current elicited by Ang (1-7) was significantly greater in Q111 cells than in wild-type Q7 cells. The effect of Ang (1-7) in mutant huntingtin-expressing cells is related to the activation of Mas receptors because A-799 abolished the effect of the heptapeptide on potassium current. Evidence is available that the Mas receptor is found in various areas of the brain, including the hippocampus, amygdala, forebrain, piriform cortex, olfactory bulb, thalamus, and portions of the hypothalamus (40, 41) and that Ang (1-7) counteracts several effects of Ang II in these and other tissues (42, 43). Furthermore, Mas expression was also discovered in the monkey retina (44). The presence of Mas in areas, such as the hypothalamus, nucleus tractus solitarii, rostral, and caudal ventrolateral medulla, provides the basis for several effects produced by its agonist, Ang-(1–7), in the brain. Although the appreciable effect of Ang (1-7) on potassium current seen in mutant Q111 cells might be related to an enhanced expression of Mas receptors, further studies are needed to confirm this possibility.

Of particular interest was the finding that the effect of Ang II on potassium current in Q111 mutant cells was negligible, contrasting with the significant increase of potassium current elicited by the peptide in wild-type Q7 cells. The reason for the small effect of Ang II on mutant neurons was the appreciable decline of expression of Ang II type 1 receptors (AT1R) found in the cell membrane of these cells. Consistent with this finding is the observation that AT1R levels are significantly decreased in postmortem putamen of HD patients relative to control individuals (45). This finding seems to be in accord with recent studies showing that the immune system plays an important role in HD and that the presence of T cell activating autoantibodies against AT1R described in all stages of the disease (21) may explain the decline in AT1R levels found in HD patients. Not only AT1R seem altered in HD but also the activity of the ACE is reduced. Studies performed in different regions of the calf and human brains with HD indicated a decline of ACE activity including a reduction of 83–92% in the globus pallidus, while the caudate and putamen of choreic patients display 62–69% decrease in the enzyme activity (25). These observations support the view that the ACE/Ang II/AT1 receptor axis is reduced in animal models and in patients with HD.

Evidence is available that independent of the conventional renin–angiotensin aldosterone (RAAS) system, different components of RAAS are expressed in cells of the cardiovascular system and play an important role on cardiovascular pathophysiology (26–33). Previous observations revealed that AT1R are present inside neurons near mitochondria leading to the hypothesis that endogenous or internalized Ang II can alter mitochondrial function and generate oxidative stress (35) (see also (34)). The finding that intracellular Ang II reduces the potassium current in wild-type Q7 cells indicate that the peptide has an intracrine action while in mutant Q111 cells the effect of intracellular Ang II was negligible, possibly due to a decreased expression of AT1R inside the cell. Of particular interest was the observation that intracellular Ang (1-7) reduced the potassium current in mutant cells, an effect opposite to that seen when the heptapeptide was added to the wild-type cells. The significance of these preliminary findings and their possible implications for HD are not known. Mutant huntingtin associates directly with brain mitochondria (46–48) disrupts calcium homeostasis, reduces ATP generation, and inhibits the mitochondrial trafficking (49). In mutant huntingtin-expressing Q111 cells, a significant increase in mitochondrial-generated superoxide is observed compared to wild-type cells (50). Moreover, mitochondrial bioenergetics and spare respiratory capacity are significantly reduced in immortalized mutant huntingtin-expressing mouse Q111 cells compared to wild-type cells (50). Thus, it is possible that mitochondrial dysfunction and increased levels of mitochondrial reactive oxygen species (ROS) may enhance the ACE2/Ang(1-7)/Mas axis in Q111 cells. Interestingly, blockade of the AT1R inhibits cardiac hypertrophy by induction of ACE2 gene expression, suppression of ROS generation, and enhancement of the ACE2/Ang(1-7)/Mas axis (51).

Electrophysiological studies performed on striatal neurons from Q175 mouse model of HD revealed that the frequency of spontaneous and miniature excitatory postsynaptic currents (EPSCs) was decreased while the frequency of spontaneous inhibitory postsynaptic currents was enhanced (52). Since Ang II reduces the potassium current and depolarizes the wild-type Q7 cells, it is possible that the lack of effect of Ang II on potassium current in mutant Q111 cells represents a compensatory mechanism against the decrease of EPSC described in striatal neurons in animal models of the disease.

## Conclusion

The present observations indicate: (1) a significant decline in the expression of Ang II AT1R in mutant Q111 cells and a drastic decrease in the effect of the peptide on potassium current; (2) a large effect of Ang (1-7) of potassium current in mutant cells, an effect suppressed by a Mas receptor antagonist. These observations indicate that the ACE2/Ang (1-7)/Mas receptor axis of the RAS in mutant neurons is predominantly activated over the ACE/Ang II/AT1R axis. Although we do not know the pathophysiological significance of these results, they support the view that the RAS is involved in the derangement of brain function seen in different degenerative diseases.

## Author Contributions

Conception and design was performed by WDM and SAP. The electrophysiological studies and the organization of the manuscript were performed by WDM tissue culture studies and revision of the manuscript were performed by SAP and flow cytometry analysis and revision of the manuscript were performed by YG.

## Conflict of Interest Statement

The authors declare that the research was conducted in the absence of any commercial or financial relationships that could be construed as a potential conflict of interest. The reviewer, PA, and handling editor declared their shared affiliation, and the handling editor states that the process nevertheless met the standards of a fair and objective review.

## References

[1] Harper P Huntington’s Disease. London: Saunders (1991).

[2] Huntington’s Disease Collaborative Research Group. A novel gene containing a trinucleotide repeat that is unstable on Huntington’s disease chromosomes. Cell (1993) 72:971–83. 10.1016/0092-8674(93)90585-E 8458085

[3] Vonsattel JP Myers RH Stevens TJ Ferrante RJ Bird ED Richardson EP Jr. Neuropathological classification of Huntington’s disease. J Neuropathol Exp Neurol (1985) 44(6):559–77. 10.1097/00005072-198511000-00003 2932539

[4] Vonsattel JP DiFiglia M Huntington’s disease. J Neuropathol Exp Neurol (1998) 57(5):369–84. 10.1097/00005072-199805000-00001 9596408

[5] Humphries ESA Dart C. Neuronal and cardiovascular potassium channels as therapeutic drug targets: promise and pitfalls. J Biomol Screen (2015) 20(9):1055–73. 10.1177/1087057115601677 26303307 PMC4576507

[6] Klapstein GJ Fisher RS Zanjani H Cepeda C Jokel ES Chesselet MF Electrophysiological and morphological changes in striatal spiny neurons in R6/2 Huntington’s disease transgenic mice. J Neurophysiol (2001) 86:2667–77. 11731527 10.1152/jn.2001.86.6.2667

[7] Miller BR Walker AG Barton SJ Rebec GV Dysregulated neuronal activity patterns implicate corticostriatal circuit dysfunction in multiple rodent models of Huntington’s disease. Front Syst Neurosci (2011) 5:26 10.3389/fnsys.2011.00026 21629717 PMC3100808

[8] Arianao MA Cepeda C Calvert CR Flores-Hernandez J Hernandez-Echegaray E Klapstein GJ Striatal potassium channel dysfunction in Huntington’s disease transgenic mice. J Neurophysiol (2005) 93:2567–74. 10.1152/jn.00791.2004 15625098

[9] Zhu D Shi J Zhang Y Wang B Liu W Chen Z Central angiotensin II stimulation promotes beta amyloid production in Sprague Dawley rats. PLoS One (2011) 6(1):e16037 10.1371/journal.pone.0016037 21297982 PMC3030571

[10] Tian M Zhu D Xie W Shi J. Central angiotensin II-induced Alzheimer-like tau phosphorylation in normal rat brains. FEBS Lett (2012) 586(20):3737–45. 10.1016/j.febslet.2012.09.004 22982863

[11] Jiang T Zhang Y-D Zhou J-S Zhu X-C Tian Y-Y Zhao H-D Angiotensin-(1-7) is reduced and inversely correlates with Tau hyperphosphorylation in animal models of Alzheimer’s disease. Mol Neurobiol (2015) 53(4):2489–97. 10.1007/s12035-015-9260-9 26044748

[12] Mertens B Vanderheyden P Michotte Y Sarre S. The role of the central renin-angiotensin system in Parkinson’s disease. J Renin Angiotensin Aldosterone Syst (2010) 11(1):49–56. 10.1177/1470320309347789 19861346

[13] Mungall BA Shinkel TA Sernia C. Immunocytochemical localization of angiotensinogen in the fetal and neonatal rat brain. Neuroscience (1995) 67:505–24. 10.1016/0306-4522(95)00044-J 7675182

[14] Palkovits M Mezey E Fodor M Ganten D Bahner U Geiger H Neurotransmitters and neuropeptides in the baroreceptor reflex arc: connections between the nucleus of the solitary tract and the ventrolateral medulla oblongata in the rat. Clin Exp Hypertens (1995) 17:101–13. 10.3109/10641969509087058 7735261

[15] Paton JF Wang S Polson JW Kasparov S. Signalling across the blood brain barrier by angiotensin II: novel implications for neurogenic hypertension. J Mol Med (2008) 86:705–10. 10.1007/s00109-008-0324-4 18443753

[16] Lind RW Swanson LW Ganten D Organization of angiotensin II immunoreactive cells and fibers in the rat central nervous system. An immunohistochemical study. Neuroendocrinology (1985) 40:2–24. 3969196 10.1159/000124046

[17] Pickel VM Chan J. Co-localization of angiotensin II and gamma-aminobutyric acid in axon terminals in the rat subfornical organ. Neurosci Lett (1995) 193:89–92. 10.1016/0304-3940(95)11673-K 7478166

[18] Ganten D Fuxe K Phillips MI Mann JFE Ganten U The brain renin-angiotensin system: biochemistry, localization, and possible role in drinking and blood pressure regulation. In: Ganong WF Martini L, editors. Frontiers in Neuroendocrinology. New York, NY: Raven (1978). p. 61–99.

[19] Hariharan A Shetty S Shirole T Jagtap AG Potential of protease inhibitor in 3-nitropropionic acid induced Huntington’s disease like symptoms: mitochondrial dysfunction and neurodegeneration. Neurotoxicology (2014) 45:139–48. 10.1016/j.neuro.2014.10.004 25445565

[20] Wang J Ho L Chen L Zhao Z Zhao W Qian X Valsartan lowers brain beta-amyloid protein levels and improves spatial learning in a mouse model of Alzheimer disease. J Clin Invest (2007) 117(11):3393–402. 10.1172/JCI31547 17965777 PMC2040315

[21] Lee DH Heidecke H Schröder A Friedemann P Wachter R Hoffmann R Increase of angiotensin II type 1 receptor auto-antibodies in Huntington’s disease. Mol Neurodegener (2014) 9:49 10.1186/1750-1326-9-49 25398321 PMC4246494

[22] Tai YF Pavese N Gerhard A Tabrizi SJ Barker RA Brooks DJ Microglial activation in presymptomatic Huntington’s disease gene carriers. Brain (2007) 130:1759–66. 10.1093/brain/awm044 17400599

[23] Bjorkqvist M Wild EJ Thiele J Andre R Lahiri N Raibon E A novel pathogenic pathway of immune activation detectable before clinical onset in Huntington’s disease. J Exp Med (2008) 205:1869–77. 10.1084/jem.20080178 18625748 PMC2525598

[24] Pavese N Gerhard A Tai YF Ho AK Turkheimer F Barker RA Microglial activation correlates with severity in Huntington disease: a clinical and PET study. Neurology (2006) 66:1638–43. 10.1212/01.wnl.0000222734.56412.17 16769933

[25] Arregui A Bennett JP Jr Bird ED Yamamura HI Iversen LL Snyder SH Huntington’s chorea: selective depletion of activity of angiotensin converting enzyme in the corpus striatum. Ann Neurol (1977) 2(4):294–8. 10.1002/ana.410020406 214022

[26] De Mello WC. Is an intracellular renin-angiotensin system involved in control of cell communication in heart? J Cardiovasc Pharmacol (1994) 23(4):640–6. 10.1097/00005344-199404000-00018 7516016

[27] De Mello WC Frohlich ED. Clinical perspectives and fundamental aspects of local cardiovascular and renal renin-angiotensin systems. Front Endocrinol (2014) 5:16. 10.3389/fendo.2014.00016 24600438 PMC3928588

[28] De Mello WC Danser AH. Angiotensin II and the heart: on the intracrine renin-angiotensin system. Hypertension (2000) 35(6):1183–8. 10.1161/01.HYP.35.6.1183 10856260

[29] De Mello WC On the pathophysiological implications of an intracellular renin receptor. Circ Res (2006) 99(12):1285–6. 10.1161/01.RES.0000253141.65450.fc 17158342

[30] Kurdi M De Mello WC Booz GW. Working outside the system: an update on the unconventional behavior of the renin-angiotensin system components. Int J Biochem Cell Biol (2005) 37:1357–67. 10.1016/j.biocel.2005.01.012 15833268

[31] De Mello WC. Influence of intracellular renin on heart cell communication. Hypertension (1995) 25:1172–7. 10.1161/01.HYP.25.6.1172 7768559

[32] Cook JL Re RN. Lessons from in vitro studies and a related intracellular angiotensin II transgenic mouse model. Am J Physiol Regul Integr Comp Physiol (2012) 302:R482–93. 10.1152/ajpregu.00493.2011 22170617 PMC3311520

[33] Kumar R Thomas CM Yong QC Chen W Baker KM. The intracrine renin-angiotensin system. Clin Sci (Lond) (2012) 123:273–84. 10.1042/CS20120089 22590974 PMC3965264

[34] Deliu E Brailoiu GC Eguchi S Hoffman NE Rabinowitz JE Tilley DG Direct evidence of intracrine angiotensin II signaling in neurons. Am J Physiol Cell Physiol (2014) 306(8):C736–44. 10.1152/ajpcell.00131.2013 24401846 PMC3989719

[35] Huang J Hara Y Anrather J Speth RC Iadecola C Pickel VM. Angiotensin II subtype 1A (AT1A) receptors in the rat sensory vagal complex: subcellular localization and association with endogenous angiotensin. Neuroscience (2003) 122:21–36. 10.1016/S0306-4522(03)00606-7 14596846

[36] Zimmerman MC Lazartigues E Lang JA Sinnayah P Ahmad IM Spitz DR Superoxide mediates the actions of angiotensin II in the central nervous system. Circ Res (2002) 91:1038–45. 10.1161/01.RES.0000043501.47934.FA 12456490

[37] Schumacker PT Angiotensin II signaling in the brain: compartmentalization of redox signaling? Circ Res (2002) 91:982–4. 10.1161/01.RES.0000045655.34731.B6 12456482

[38] De Mello WC. Chemical communication between heart cells is disrupted by intracellular renin and angiotensin II: implications for heart development and disease. Front Endocrinol (2015) 6:72. 10.3389/fendo.2015.00072 26042086 PMC4437035

[39] Gwathmey TM Alzayadneh EM Pendergrass KD Chappell MC. Novel roles of nuclear angiotensin receptors and signaling mechanisms. Am J Physiol Regul Integr Comp Physiol (2012) 302(5):R518–30. 10.1152/ajpregu.00525.2011 22170620 PMC3311515

[40] Young D O’Neill K Jessell T Wigler M. Characterization of the rat mas oncogene and its high-level expression in the hippocampus and cerebral cortex of rat brain. Proc Natl Acad Sci U S A (1988) 85:5339–42. 10.1073/pnas.85.14.5339 2455902 PMC281746

[41] Bunnemann B Fuxe K Metzger R Mullins J Jackson TR Hanley MR Autoradiographic localization of mas proto-oncogene mRNA in adult rat brain using in situ hybridization. Neurosci Lett (1990) 114:147–53. 10.1016/0304-3940(90)90063-F 2203997

[42] Ferrario CM Chappell MC Tallant EA Brosnihan KB Diz DI Counterregulatory actions of angiotensin (1-7). Hypertension (1997) 30:535–411. 10.1161/01.HYP.30.3.535 9322978

[43] De Mello WC Ferrario CM Jessup JA. Beneficial versus harmful effects of Angiotensin (1-7) on impulse propagation and cardiac arrhythmias in the failing heart. J Renin Angiotensin Aldosterone Syst (2007) 8(2):74–80. 10.3317/jraas.2007.015 17703433

[44] Kitaoka T Sharif M Hanley MR Hjelmeland LM. Expression of the MAS proto-oncogene in the retinal pigment epithelium of the rhesus macaque. Curr Eye Res (1994) 13:345–51. 10.3109/02713689409167298 8055698

[45] Ge J Barnes NM Alterations in angiotensin AT1 and AT2 receptor subtype levels in brains regions from patients with neurodegenerative disorders. Eur J Pharmacol (1996) 297:299–306. 10.1016/0014-2999(95)00762-8 8666063

[46] Panov A Gutekunst C Leavitt B Hayden M Burke J Strittmatter W Early mitochondrial calcium defects in Huntington’s disease are a direct effect of polyglutamines. Nat Neurosci (2002) 5:731–6. 10.1038/nn884 12089530

[47] Choo YS Johnson GV MacDonald M Detloff PJ Lesort M. Mutant huntingtin directly increases susceptibility of mitochondria to the calcium-induced permeability transition and cytochrome c release. Hum Mol Genet (2004) 13:1407–20. 10.1093/hmg/ddh162 15163634

[48] Petrassch-Parwez E Nguyen H Lobbecke-Schumacher M Habbes H Wieczorek S Riess O Cellular and subcellular localization of Huntingtin aggregates in the brain of a rat transgenic for Huntington disease. J Comp Neurol (2007) 501:716–30. 10.1002/cne.21272 17299753

[49] Orr A Li S Wang C-E Li H Wang J Rong J N-terminal mutant huntingtin associates with mitochondria and impairs mitochondrial trafficking. J Neurosci (2008) 28:2783–92. 10.1523/JNEUROSCI.0106-08.2008 18337408 PMC2652473

[50] Siddiqui A Rivera-Sánchez S Castro Mdel R Acevedo-Torres K Rane A Torres-Ramos CA Mitochondrial DNA damage is associated with reduced mitochondrial bioenergetics in Huntington’s disease. Free Radic Biol Med (2012) 53:1478–88. 10.1016/j.freeradbiomed.2012.06.008 22709585 PMC3846402

[51] Tanno T Tomita H Narita I Kinjo T Nishizaki K Ichikawa H Olmesartan inhibits cardiac hypertrophy in mice overexpressing renin independently of blood pressure: its beneficial effects on ACE2/Ang(1-7)/Mas axis and NADPH oxidase expression. J Cardiovasc Pharmacol (2016) 67(6):503. 10.1097/FJC.0000000000000374 26886190

[52] Indersmitten T Tran CH Cepeda C Levine MS Altered excitatory and inhibitory inputs to striatal medium-sized spiny neurons and cortical pyramidal neurons in the Q175 mouse model of Huntington’s disease. J Neurophysiol (2015) 113(7):2953–66. 10.1152/jn.01056.2014 25673747 PMC4416625

